# Prevalence of myalgic encephalomyelitis/chronic fatigue syndrome (ME/CFS) in Australian primary care patients: only part of the story?

**DOI:** 10.1186/s12889-022-13929-9

**Published:** 2022-08-09

**Authors:** Nneka Orji, Julie A. Campbell, Karen Wills, Martin Hensher, Andrew J. Palmer, Melissa Rogerson, Ryan Kelly, Barbara de Graaff

**Affiliations:** 1 Menzies Institute for Medical Research, University of Tasmania, Hobart, Australia; 2 School of Computing & Information Systems, University of Melbourne, Melbourne, Australia

**Keywords:** Chronic fatigue syndrome, Myalgic encephalomyelitis, ME/CFS, Prevalence, Primary care, Mixed methods

## Abstract

**Background:**

ME/CFS is a disorder characterized by recurrent fatigue and intolerance to exertion which manifests as profound post-exertional malaise. Prevalence studies internationally have reported highly variable results due to the 20 + diagnostic criteria. For Australia, the prevalence of ME/CFS based on current case definitions is unknown.

**Objectives:**

To report prevalence of ME/CFS in patients aged ≥ 13 years attending Australian primary care settings for years 2015–2019, and provide context for patterns of primary care attendance by people living with ME/CFS.

**Methodology:**

Conducted in partnership with the Patient Advisory Group, this study adopted a mixed methods approach. De-identified primary care data from the national MedicineInsight program were analyzed. The cohort were regularly attending patients, i.e. 3 visits in the preceding 2 years. Crude prevalence rates were calculated for years 2015–2019, by sex, 10-year age groups, remoteness and socioeconomic status. Rates are presented per 100,000population (95% confidence intervals (CI)). Qualitative data was collected through focus groups and in-depth 1:1 interview.

**Results:**

Qualitative evidence identified barriers to reaching diagnosis, and limited interactions with primary care due to a lack of available treatments/interventions, stigma and disbelief in ME/CFS as a condition.

In each year of interest, crude prevalence in the primary care setting ranged between 94.9/100,000 (95% CI: 91.5–98.5) and 103.9/100,000 population (95%CI: 100.3–107.7), equating to between 20,140 and 22,050 people living with ME/CFS in Australia in 2020. Higher rates were observed for age groups 50-59 years and 40-49 years. Rates were substantially higher in females (130.0–141.4/100,000) compared to males (50.9–57.5/100,000). In the context of the qualitative evidence, our prevalence rates likely represent an underestimate of the true prevalence of ME/CFS in the Australian primary care setting.

**Conclusion:**

ME/CFS affects a substantial number of Australians. Whilst this study provides prevalence estimates for the Australian primary care setting, the qualitative evidence highlights the limitations of these. Future research should focus on using robust case ascertainment criteria in a community setting. Quantification of the burden of disease can be used to inform health policy and planning, for this understudied condition.

**Supplementary Information:**

The online version contains supplementary material available at 10.1186/s12889-022-13929-9.

## Introduction

Myalgic Encephalomyelitis/Chronic Fatigue Syndrome (ME/CFS) is a condition with recurrent fatigue and intolerance to exertion which mainly manifests as profound or pathological post-exertional malaise [1, 2]. Previously, the WHO had interchangeably used ME and CFS, and classified ME as a neurological disorder [3]. Recent studies have reported broader systemic and empirical classifications for ME/CFS [4, 5]. Whilst new empirical evidence continues to emerge regarding the possible causal factors and manifestations of ME/CFS [4, 6] there remains substantive evidence gaps on the aetiology of ME/CFS. The condition is characterized by multiple symptoms including post-exertional malaise, severe and recurrent fatigue, headaches, digestive disorders, cardiac symptoms, and cognitive impairment, all of which adversely impact on routine functioning and wellbeing of individuals [7–9].

The broad array of symptoms, unclear and limited understanding of the aetiology are critical factors that have contributed to the development of more than 20 + diagnostic criteria for ME/CFS over recent decades [10–12]. Currently, the most widely accepted criteria include the Institute of Medicine (IOM) criteria [4]; the International Consensus Criteria (ICC) [5]; and the Canadian Consensus Criteria (CCC) [13]. The CCC recognizes and differentiates cases by clinical manifestations and symptoms whilst the ICC has a broader spectrum of symptoms to guide a more in-depth case definition. The IOM criteria, which were proposed in 2015, focuses more on the central characteristics which manifest as physical illnesses [4, 6]. Other diagnostic criteria that have been used include the Fukuda criteria, Holmes, Oxford, Australia, and Ramsay criteria [11]. It is important to note that these criteria were developed with different aims, some having been derived for CFS, others for ME, and others for ME/CFS; and for different purposes: clinical diagnosis or case ascertainment in research.

In turn, the array of diagnostic criteria has contributed to a lack of comparable prevalence data. For instance, in a UK study set in primary care, different diagnostic criteria provided different rates: Fukuda 0.19%; CCC 0.11%; Epidemiological Case Definition 0.03% [7]. The variability of prevalence estimates was described in a recent systematic review [11]*.* A meta-analysis of prevalence estimates was undertaken, yielding an estimate of 0.68% (95%CI: 0.48–0.97), however, heterogeneity was very high (*I*^*2*^ = 99.4%). Prevalence estimates were also reported by case definitions, with estimates ranging between 0.34% using the Holmes criteria and 2.52% using the Australian criteria. The Australian criteria were used in a 1990 study [14] although this no longer reflects current understanding of the condition. To-date, this is the only prevalence study conducted in Australia, therefore the primary aim of this present study is to estimate the prevalence of ME/CFS in the Australian primary care setting.

## Methods

### Patient advisory group

Prior to commencement of this study, the research team began working with a 20-member Patient Advisory Group. The aim of this was to ensure the voices of patients were included and their insights reflected in all stages of this study. The feedback provided was that many people living with ME/CFS do not attend general practitioners (GPs; i.e. primary care physicians) for ME/CFS-related care due to a lack of treatment(s) and stigma/disbelief associated with the condition. To address the potential under-estimation of prevalence due to such factors, we conducted focus groups and long interviews with ME/CFS patients to further understand patterns of GP attendance. As such, this study will present crude prevalence estimates generated from a nationally representative primary care dataset along with qualitative data to provide greater context for these estimates.

### Quantitative analysis

#### Setting and study population

The datasets for this study were extracted from the MedicineInsight database, which is managed by NPS MedicineWise, an independent, not-for-profit organization [15, 16]. The database contains de-identified patient data from primary care/general practices across Australia which has been described elsewhere [16]. The patients included in this database are similar in terms of age, sex and socio-economic status to all Australian patients who have received at least one general practitioner (GP) Medicare Benefits Schedule (MBS) subsidized consultation (i.e., the universal health insurance program) [17].

A unique patient identifying number allows patients within practices to be tracked over time to produce longitudinal data. As of 31/12/2019, 671 general practices were participating, with > 2.2 million active patients.

The study period was defined as 1^st^ January 2014 to 31^st^ December 2019, and we defined patients using observable person-time [18]. The study population was defined as those meeting the following criteria:

- Visited a practice site and met specific MedicineInsight data quality requirements.
- At least one clinical encounter with the practice, including face-to-face or phone call during the study period. A lookback period was adopted for clinical encounters, see section below for details.
- Have valid information for age (aged ≥13 years in each year of interest).
- Observable person-time during the study period commenced from the patient’s first recorded clinical encounter at the practice and ended at their last recorded clinical encounter with a one-year ‘lookback’ period to improve case ascertainment [18].

#### Case definition

In addition to the criteria above, patients were included as cases if they had:

- 1. A diagnosis of “myalgic encephalomyelitis”, “chronic fatigue syndrome”, “ME”, “CFS”, or “ME/CFS”. Algorithms were used to identify coded and free-text information with the key words in the fields of either ‘encounter_reason’, or ‘diagnosis_reason’
- 2. Encounter/diagnosis of ME/CFS from the first lookback year (01/01/2014) up to 31/12/2019.

Records of ‘chronic fatigue’ were excluded as this symptom alone does not provide evidence of an ME/CFS diagnosis.

#### Lookback period and case definition assumptions

**Fig. 1:**
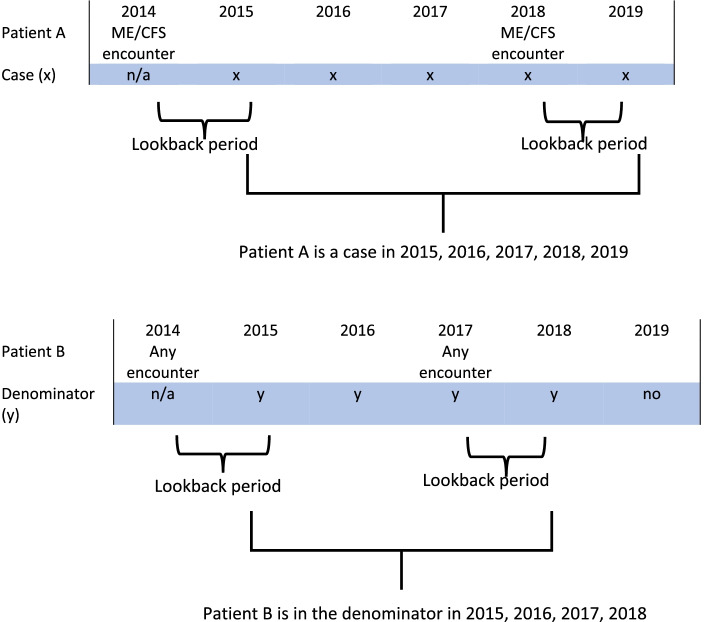
Definition of Observable time [18]. Adapted from Rassen et al., (2019) Clinical epidemiology

Based on discussions with the Patient Advisory Group, a 12-month lookback period was adopted to improve case ascertainment. This was based on the understanding that some ME/CFS patients do not attend a GP clinic for ME/CFS-related problems on an annual basis. Therefore, the prevalence estimates for 2015 includes all ME/CFS encounters that occurred from 01/01/2014 to 31/12/2015. Based on the focus groups discussions and feedback from the Patient Advisory Group, assumptions were also made that in the situation that a patient had ME/CFS encounters several years apart, they would be considered a prevalent case for all years in between the encounters. For example (see Fig. 1), if a patient had an ME/CFS encounter in 2014 and then again in 2018, they will be considered a case in:

- 2015 (due to the lookback period, i.e. 2014);
- 2016 and 2017.
- 2018 (as they had an encounter in 2018)
- 2019 (due to the lookback period, i.e., 2018).

In this example, for both 2016 and 2017, we assumed that the patient remains an ME/CFS case, reflecting the chronic nature of the condition and the very unclear and limited possibility of patient recovery from ME/CFS within the given period under review.

### Statistical analysis

Data were analysed in the Secure Unified Research Environment (SURE), a highly secure virtual environment, using STATA software (version 16, Stata Corp. College Station, TX, USA). Descriptive statistics were calculated for cases for each period of interest from 2015 through to 2019. For each year of interest, descriptive characteristics of the cohort are presented as means (standard deviations (SD)) for normally distributed continuous variables, along with medians (interquartile ranges (IQR)), and frequency and percentages as appropriate.

Annual crude prevalence rates per 100,000 persons (95% CI) were calculated for each year of interest, i.e. 2015–2019, along with rates by sex, 10-year age groups, Index of Relative Socio-economic Advantage and Disadvantage (IRSAD) quintiles, and remoteness areas as measured by the Australian Bureau of Statistics’ Australian Statistical Geography Standards [19]. As MedicineInsight data is representative of the Australian primary care patient cohort, age standardization was deemed unnecessary [15].

### Qualitative analysis

#### Validated guidelines, information power and ethics

We adopted qualitative research methods to supplement and augment the quantitative analysis by providing deeper contextualization and nuance to the quantitative findings [20]. ‘Mixed methods’ research capitalizes on the strengths of quantitative and qualitative data within a single study by integrating the two data types [21, 22]. We used the Standards for Reporting Qualitative Research that is a list of 21 items considered essential for complete transparent reporting of qualitative research and the Consolidated Criteria for Reporting Qualitative Research for focus groups and interviews [23].

We adopted the theory of ‘information power’ when constructing the size and representation of the focus groups and the number of interviews [24]. Information power is underpinned by a negative association between the depth of information provided by the participant(s) and the number of participants required. That is, a high level of information by a small number of participants provides strong qualitative evidence, particularly where data saturation is achieved, as in our study. More specifically, information power of an interview sample is determined by items such as: study aim; sample specificity; use of established theory; quality of dialogue; and analysis strategy [24]. Quality of dialogue was important for our sample as informed by our discussions with the Patient Advisory Group. The group indicated strong dialogue was expected during the investigation of the relatively narrow study aim for this current study of establishing prevalence in Australian primary care patients. To generate discussion amongst age demographics with similar lived experience, focus groups were stratified into the age groups of older, middle-aged, and younger people with ME/CFS. Carers were allocated to a separate focus group to also facilitate strong dialogue with shared experience.

#### Recruitment

Participants were recruited from an advertisement on Emerge Australia’s website, radio, and newspaper advertising, and ME/CFS Facebook platforms to ensure a wider reach. Inclusion criteria were a self-reported diagnosis of ME/CFS and aged 18 years or older. People who indicated their interest in the study were provided with a detailed information sheet and consent form. A pre-screening interview was then conducted by telephone (5–10 min) to answer questions, assess eligibility for the study, collect informed written consent and socio-demographic information for the recruitment database that would be used for sampling of focus groups and individual interviews (email address; age; sex; postcode; time of onset of first symptoms; time of diagnosis).

#### Data gathering and analysis

As part of the broader qualitative study regarding ME/CFS in Australia, we used semi-structured focus groups triangulated with both long interviews and the quantitative analysis to also investigate timing, barriers, and enablers to diagnosis of ME/CFS in the Australian primary healthcare setting. Focus groups were conducted virtually using computer assisted meeting technology to simulate a focus group environment and were audio recorded and transcribed verbatim with identifying material (names) removed. Semi -structured questionnaires were used to guide the discussion with each focus group and the same interviewers and observers participated in the focus groups and interviews. Focus group data were triangulated with individual interviews to explore emergent themes [25]. Given that debilitating fatigue is a key symptom of the chronic condition, two focus group interviews (of the same focus group) of no longer than 45 min each were conducted with the opportunity to also provide free text responses between the focus groups (2 days) to the questions that guided the interviews.

Thematic analysis was conducted inductively [26, 27] with the assistance of NVivo software. Emergent themes were discussed between co-authors and the Patient Advisory Group. Reflective notes (by the interviewers and observers) and meeting notes (for example with the Patient Advisory Group) were kept.

### Ethics approval

Ethics approval for the quantitative study was granted by the University of Tasmania’s Health and Medical Research Ethics Committee (H0018473) and the qualitative component of the through the University of Tasmania (H0018683). Approval to conduct this study was granted by the MedicineInsight independent Data Governance Committee (reference number: 2019–026).

## Results

### Quantitative analysis

#### Demographic characteristics of the study population

The demographic characteristics for the overall study population are presented in Table 1. In each, the majority of cohort was female (55.3–55.4%) and almost two-thirds resided in a major city (62.1–64.2%). For IRSAD quintiles, the proportion of the cohort in quintile 1 (i.e. the most disadvantaged) was the consistently the lowest (15.8–16.8%) and highest for quintile 5 (most advantaged: 23.5–24.2%).

**Table 1:** Characteristics of study population: 2015–2019

	2015	2016	2017	2018	2019
Number of patients	2,811,876 % (n)	2,939,792 % (n)	3,019,638 % (n)	3,043,918 % (n)	2,991,489 % (n)
Sex % (n):					
Male	44.7 (1,255,682)	44.7 (1,314,191)	44.7 (1,349,991)	44.7 (1,359,649)	44.6 (1,333,521)
Female	55.3 (1,556,194)	55.3 (1,625,601)	55.3 (1,669,647)	55.3 (1,684,269)	55.4 (1,657,968)
Age groups % (n):					
13–19	9.2 (258,375)	9.1 (268,042)	9.0 (272,853)	8.9 (272,198)	8.8 (263,163)
20–29	16.8 (473,049)	16.9 (497,340)	16.9 (510,591)	16.8 (512,067)	16.7 (498,436)
30–39	17.3 (485,354)	17.5 (513,357)	17.6 (531,723)	17.8 (541,850)	17.9 (534,827)
40–49	16.3 (457,570)	16.1 (472,948)	15.9 (480,509)	15.7 (477,754)	15.5 (462,430)
50–59	14.9 (419,465)	14.7 (432,341)	14.5 (438,992)	14.4 (437,957)	14.3 (426,778)
60–69	12.6 (353,291)	12.5 (367,427)	12.4 (375,807)	12.5 (379,266)	12.6 (375,879)
70–79	7.78 (218,777)	8.0 (235,805)	8.3 (251,259)	8.6 (261,238)	8.9 (267,291)
80 +	5.2 (145,995)	5.2 (152,532)	5.2 (157,904)	5.3 (161,588)	5.4 (162,685)
Remoteness indicator: %(n)					
Major City	62.1 (1,745,669)	62.8 (1,845,847)	63.3 (1,912,829)	63.8(1,942,453)	64.2 (1,919,721)
Inner regional	24.6 (692,835)	24.2 (711,228)	23.8 (719,996)	23.6 (719,387)	23.5 (704,270)
Outer regional	11.3 (318,701)	11.0 (322,066)	10.7 (324,164)	10.5 (320,768)	10.3 (309,540)
Remote/very Remote	1.9 (54,671)	2.1 (60,651)	2.1 (62,649)	2.0 (61,310)	1.9 (57,958)
IRSAD quintiles % (n):					
1(most disadvantaged)	16.8 (471,802)	16.4 (481,149)	16.2 (487,922)	16.0 (488,421)	15.8 (473,940)
2	18.3 (513,368)	18.0 (530,242)	17.9 (540,377)	17.7 (539,537)	17.6 (527,685)
3	21.5 (604,492)	21.8 (640,833)	22.1 (667,073)	22.5 (677,415)	22.4 (669,526)
4	19.3 (542,827)	19.8 (582,249)	20.1 (607,076)	20.4 (620,508)	20.7 (618,097)
5(most advantaged)	24.2 (679,387)	24.0 (705,319)	23.8 (717,190)	23.6 (718,037)	23.5 (702,241)

#### Demographic characteristics of ME/CFS cases

Table 2 provides an overview of the number of cases identified in each period (year) of interest and the demographic characteristics for each year from 2015 to 2019. The number of cases identified for each year from 2015 to 2019 ranged between 2,841 and 3,056, with three-quarters being female in each year. The demographic characteristics of cases for each year are provided in Table 2 by age, remoteness indicator and IRSAD quintiles. Approximately one-fifth of cases were in age groups 30–39, 40–49 and 50 -59 years respectively over the years under review. Almost three-fifths of cases resided in a major city for all the years under review, and for IRSAD quintiles, one-quarter of cases were observed for both the 5^th^ quintile (most advantaged) and third quintile in each year respectively.

**Table 2:** ME/CFS prevalent cases and demographic characteristics: 2015–2019

	2015	2016	2017	2018	2019
Number of cases	2,854 % (n)	3,056 % (n)	3,010 % (n)	2,959 % (n)	2,841 % (n)
Sex % (n):					
Male	25.4 (722)	24.8 (756)	25.7 (769)	24.7 (729)	23.9 (680)
Female	74.6 (2,130)	75.2 (2,298)	74.3 (2,237)	75.2 (2,226)	76.1 (2,158)
Mean age (range)	44.1 years (13–93)	44.2 years (13–94)	44.6 years (13–93)	44.7 years (13–93)	44.9 years (13- 93)
Age groups % (n):					
13–19	7.3 (207)	6.6 (203)	7.3 (195)	6.2 (183)	6.1 (176)
20–29	15.6 (444)	15.7 (478)	15.6 (478)	16.0 (474)	15.8 (449)
30–39	18.0 (517)	18.6 (568)	18.1 (545)	17.6 (520)	17.9 (511)
40–49	19.0 (543)	19.1 (583)	19.0 (560)	19.5 (576)	19.6 (559)
50–59	21.0 (598)	20.1 (613)	20.1 (610)	19.7 (582)	18.9 (536)
60–69	12.8 (362)	13.6 (415)	12.8 (429)	14.5 (429)	14.3 (405)
70–79	5.4 (150)	5.4 (165)	5.4 (163)	5.9 (166)	6.0 (171)
80 +	1.2 (31)	0.9 (29)	1.2 (30)	0.9 (28)	1.2 (34)
Remoteness indicator: %(n)	*n* = 2,836	*n* = 3,034	*n* = 2,985	*n* = 2,943	*n* = 2,831
Major City	56.8 (1,611)	58.6 (1,777)	\58.4 (1,744)	58.0 (1,708)	58.3 (1,651)
Inner regional	30.5 (863)	29.8 (904)	28.7 (858)	28.9 (851)	29.1 (823)
Outer regional	11.9 (339)	10.7 (324)	11.8 (353)	11.9 (350)	11.3 (391)
Remote/very Remote	0.8 (23)	0.9 (29)	1.0 (30)	1.2 (34)	1.3 (38)
IRSAD quintiles % (n):	*n* = 2,838	*n* = 3,034	*n* = 2,986	*n* = 2,944	*n* = 2,832
1(most disadvantaged)	15.1 (428)	14.9 (451)	15.5 (463)	15.1 (445)	14.2 (402)
2	20.1 (569)	9.3 (586)	18.6 (556)	18.0 (530)	19.0 (539)
3	22.0 (624)	22.5 (683)	22.1 (660)	22.1 (660)	23.2 (657)
4	17.9 (509)	18.1 (548)	19.3 (575)	19.9 (586)	19.4 (550)
5(most advantaged)	24.9 (708)	5.3 (766)	24.5 (732)	24.6 (723)	24.1 (684)

#### Crude prevalence estimates: 2015 to 2019

Table 3 provides the crude prevalence estimates by age and sex for 2015 to 2019. In 2015, the crude prevalence rate was 101.5 per 100,000 population (95% CI: 97.8 – 105.3) in the primary care setting. Similar rates were reported for 2016–2019, ranging between 94.9 per 100,000 population (95%CI: 91.5–98.5) to 103.9 per 100,000 (95%CI: 100.3–107.7). In all the years under review, the crude prevalence estimates were highest for females, ranging between 141.4 per 100,000 population (95%CI: 135.7–147.3) to 130.0 per 100,000 population (95%CI: 124.7–135.8. In comparison, the crude prevalence for males ranged from 50.9 per 100,000 population (95%CI: 47.2–54.9) to 57.5 per 100,000 population (95%CI: 53.4–61.9). Crude prevalence rates were highest for age groups 50–59 years for all the years under review except for 2017 where it was highest amongst age groups 60–69 years. Similarly, rates for both females and males were higher amongst age groups 50 -59 years except in 2019 where the rates were higher in females of age group 40–49 years. Crude prevalence estimates by remoteness areas were higher for cases residing in inner regional areas, ranging between 116.9/100,000 (95%CI: 109.0–125.1) and 126.8/100,000 (95% CI: 118.7–135.4). Interestingly, for IRSAD quintiles, the lowest quintiles were observed to have the lowest prevalence whilst the highest prevalence was observed amongst the highest quintiles.

**Table 3:** Crude prevalence of ME/CFS per 100,000 persons, 2015–2019

	2015	2016	2017	2018	2019
All persons rate (95% CI)	101.5(97.8–105.3)	103.9 (100.3–107.7)	99.7 (96.7–103.3)	97.2(93.7–100.8)	94.9 (91.5–98.5)
Females	136.9 (131.1–142.8)	141.4(135.7–147.3)	133.9(128.5–139.7)	132.0(126.7–137.8)	130.0(124.7–135.8)
Males	57.5 (53.4–61.9)	57.5(53.5–61.8)	56.9(53.0–61.1)	53.6(49.8–57.7)	50.9(47.2–54.9)
Age groups:					
13–19	80.1(69.691.8)	75.7(65.7–86.9)	71.5(61.8–82.2)	67.2(57.8–77.7)	66.9(57.4–77.5)
20–29	94.0(85.5–103.2)	96.1(87.7–105.1)	93.4(85.2–102.2)	92.6(84.2–101.3)	90.1(81.9–98.8)
30–39	106.5(97.5–116.1)	110.6(101.7–120.1)	102.3(93.9–111.3)	95.8(87.7–104.4)	95.4(87.3–104.0)
40–49	118.8(109.1–129.3)	123.3(113.5–133.7)	116.3(106.9–126.4)	120.4(110.7–130.6)	120.5(110.7–130.9)
50–59	142.6(131.4–154.5)	141.8(130.81–53.5)	139.0(128.2150.4)	132.9(122.3–144.1)	125.6(115.2–136.7)
60–69	102.5(92.2–113.6)	103.0(102.4124.4)	144.2(103.6–125.5)	113.1(102.7–124.3)	107.8(97.5–118.8)
70–79	68.6(58.0–80.5)	69.9(59.7–81.5)	64.5(54.9–75.2)	63.1(53.9–73.6)	63.9(54.8–74.3)
80 +	21.2(14.4–30.1)	19.0(12.7–27.3)	19.0(12.8–27.1)	17.3(11.5–25.0)	20.9(14.5–29.2)
Remoteness area					
Major city	92.3(87.8–96.9)	96.3(91.9–100.9)	91.0(86.8—95.5)	87.8(83.7–92.1)	85.9 (81.9—90.2)
Inner regional	124.5 (116.4–133.2)	126.8 (118.7–135.4)	119.0(111.2–127.3)	118.2(110.3–126.4)	116.9(109.0–125.1)
Outer regional	106.4(95.4–118.3)	100.6(89.9–112.2)	108.9 (97.8–120.9)	109.1 (97.9–121.1)	103.1 (92.1–115.0)
Remote/very remote	42.1(26.7–63.1)	47.8 (32.0–68.7)	46.3 (31.0–66.5)	53.8 (37.0–75.6)	65.6 (46.4–89.9)
IRSAD quintiles:					
1(most disadvantaged)	90.5 (82.1- 99.5)	93.5 (85.1—102.5)	94.7 (86.3—103.7)	90.9 (82.7—99.8)	84.8 (76.7—93.5)
2	110.8 (101.9–120.3)	110.5 (101.8–119.8)	102.9 (94.5—111.8)	98.2(90.1—106.9)	101.9(93.5–110.9)
3	103.2 (95.3—111.6)	106.6 (98.7—114.8)	98.9(91.5—106.8)	97.4 (90.1—105.2)	98.1 (90.8—105.9)
4	93.7(85.8–102.3)	94.1(86.4—102.3)	94.7 (87.1—102.8)	94.4(86.9 -102.4)	88.9(81.7—96.7)
5(most advantaged)	104.1(96.5—112.0)	108.5 (100.9–116.4)	101.7 (94.4—109.3)	100.3(93.1–107.9)	109.7(101.7–118.3)

### Qualitative analysis

#### Participant characteristics

The theory of information power guided the *n* = 4 focus groups with *n* = 19 participants. For people with ME/CFS, three focus groups were stratified for age (older people with ME/CFS mean age 66 years; middle-aged people with ME/CFS mean age 40 years; younger people with ME/CFS mean age 32 years) with a purposive mix of geographical location (Australian state; capital city and rural/remote) and sex. The final focus group comprised carers who varied in age and geographical location. Follow-up long interviews were conducted (*n* = 6) with people with ME/CFS and carers to explore emerging themes in more detail.

#### Thematic analysis

 The central theme that emerged from the qualitative data was the lack of a timely diagnosis for ME/CFS in the Australian primary healthcare setting. Many people discussed the extended time in ‘years’ and seeing ‘heaps of GPs’ before their diagnosis was obtained. Participants also talked about differential diagnoses (e.g. ‘depression’) and ‘treatments’ (e.g. ‘physio’) that were suggested to people with ME/CFS before a diagnosis of ME/CFS was considered. One participant with ME/CFS talked about not obtaining a diagnosis for ‘a long time’ and feeling ‘unsupported’ and ‘disbelieved’:

*But I saw heaps of people and I didn’t get much help for a long time, and I felt very unsupported and disbelieved. And I remember one of the doctors suggesting, “Oh, maybe you should go and see a physio.” And I was like, “A physio? Why would I see a physio? What’s a physio going to do?” And I remember just bursting into tears, and that was about a year after I was really sick and nothing was happening, and all my results, like the bloods all came back normal, like everyone else’s does” (Female with ME/CFS, FG4)*

A carer of a person with ME/CFS also said that it took several years to reach a diagnosis for her daughter:

*With my daughter, it was several years, maybe three or four. She was kept being told she had depression and she said, "I don’t feel depressed”. So yeah, took a while.” (Carer, FG3)*

Selective coding of the central theme led to the subthemes of 1) the dearth of specialist primary healthcare professionals for ME/CFS particularly in rural and regional areas; 2) the prohibitive expense of accessing specialist primary healthcare professionals to reach a diagnosis; and 3) after diagnosis of ME/CFS patients may either not attend a primary healthcare physician due to lack of definitive treatment for ME/CFS or when they attend it maybe for other medical reasons other than for ME/CFS but avoiding GPs for their ME/CFS. To illustrate sub-themes 1 and 3, many participants discussed the lack of awareness of the condition in the Australian primary healthcare setting and the reluctance to recognise the condition. One participant mentioned the previous experience of a primary healthcare professional with people living with ME-CFS:

*The only ones who treated me decently were those who had some personal exposure to people with CFS. The others were next to useless - there is little worse for a medical person than a problem that they cannot solve. They hate to be shown up as not being perfect. And they communicate that to you (Female with ME/CFS, Focus Group 1)*

To illustrate subtheme 3 one focus group participant said that after being diagnosed there was “no point” due to the lack of understanding of the condition in the primary healthcare sector and that she was too tired to explain her condition to yet another doctor:

*Initially I was seeing a succession of GP’s to find a diagnosis and possible treatment. I no longer see a GP for CFS because there appears to be nothing that can be done (Female with ME/CFS, FG1)*

*Lastly, I am too tired anymore to bother trying to explain my condition to a new Doctor, for the umpteenth time (Female with ME/CFS, FG1)*

Table 4 also provides a selection of verbatim quotes to support the central and subthemes of the qualitative analysis.

**Table 4:** Examples of verbatim quotes that support the central and subthemes leading to an underestimate of prevalence of people with ME/CFS in Australia’s primary healthcare sector

Focus Group	Verbatim Quote
Focus Group 1, Female with ME/CFS	*To me the biggest issue here is that without a test for CFS we are stuck having to rule out everything else which takes a long time and then determining if you are experiencing sufficient symptoms to fit the criteria may also require some time given that the condition and the symptoms you experience fluctuate*
Focus Group 2, Female with ME/CFS	*Both the GPs I have had have been sympathetic but not knowledgeable about CFS, they have provided letters *etc. *when requested and the first filled out the forms for Centrelink, I don’t consider them to have/be treating me for CFS, I see them for other medical issues thought I may mention if these may be affecting my CFS or if I have concerns about medication they may be prescribing interacting or affecting me because of my condition. Having to educate the GP and explain things can make this more stressful even with GPs who are sympathetic*
Focus Group 1, Male with ME CFS	*I’ve had a number of GPs, and my GPs were of no use in diagnosis, but they were good in getting me to resources*
Focus Group 3, Carer of a person living with ME/CFS	*Well, it was the GP initially who kept telling my daughter that she had depression. She eventually changed GPs and also found a – we found a doctor who specialised in CFS/ME*
Focus Group 2, Male with ME/CFS	*So, it took me 18 months. I first got the ‘flu in—well, a particularly bad flu… I was like Male 1 and Female 1, peak of my fitness and working in a corporate environment and for the next 13 months—well, about 15 months, it was a living hell because I couldn’t figure out what was wrong, I’d go to GPs, no one could tell me, told me it was stress so I tried cutting back on stressful things…”*
Focus Group 2, Male with ME/CFS	*“…they finally said, “It sounds like chronic fatigue syndrome.” This was a year after I’d been sick and I’d gone to just those day clinic GPs and they didn’t have a constant GP and that wasn’t working out for obvious reasons because they didn’t have the time or the inclination to listen to the map that I was trying to draw out for them of this constellation of symptoms. I finally went to—it was wife at the time’s GP. So, my first appointment with her, she referred me to the specialist”*

In summary, our qualitative data suggested that time to diagnosis for people living with ME-CFS was a protracted process with formal diagnosis taking years and a reluctance of primary healthcare physicians to record ME-CFS in medical notes, including the recording of differential diagnosis before ME-CFS diagnosis is reached.

## Discussion

This is the first study to investigate the prevalence of ME/CFS using primary care data in Australia. This is also the first study to use mixed methods where qualitative data was triangulated with quantitative data to provide a deeper contextualization to the quantitative findings [10]. Based on primary care data, we estimated the prevalence of ME/CFS in the Australian general practice setting between 2015 and 2019 to range between 94.9/100,000 and 103.9/100,000 populations (i.e., 0.094%—0.14%). In turn, this would equate to between 20,140 and 22,050 people aged 13 years and older living with ME/CFS across Australia in 2020 [28]. The timing of our study (2015–2019) is important, as it generates estimates of ME/CFS prevalence in a period before the appearance of Long COVID and other sequelae of COVID-19 (from 2020 onwards).

Our prevalence estimates were calculated using a large dataset that is representative of the Australian general patient population in terms of age and gender. However, it is important to note that we expect these rates to be underestimates of the true prevalence of ME/CFS in the Australian general practice setting. The qualitative evidence identified barriers to reaching diagnosis leading to prolonged times to diagnosis, along with a lack of available treatments and stigma. A recently published study involving a survey of hospital-based medical doctors in the UK reported that 27% of respondents had received formal training on ME/CFS; 89% did not know how to diagnose the condition; and 93% did not feel confident working with this patient population [29]. Concerningly, 82% of respondents reported that ME/CFS was either partly of entirely a psychological condition. Whilst no similar study has been published for Australia, our qualitative evidence suggests that this may also be relevant in the Australian context.

An additional rationale for our assumption that we have underestimated prevalence is that GP encounters in the quantitative dataset do not necessarily capture an ME/CFS case when a symptom such as dizziness is recorded rather than ME/CFS. Appendix 1 contains further information regarding other potential reasons for an underestimate of prevalence.

Our results are similar to those published by Nacul and colleagues [7]. This UK-based study used primary care data and estimated prevalence using three diagnostic criteria: Centres for Disease Control (CDC) criteria: 0.19%; Canadian criteria: 0.11%; and 0.003% using Epidemiological Case definition criteria. As noted above, physicians participating in a UK study had limited knowledge of ME/CFS, potentially contributing to an underestimate of prevalence [29]. Higher rates were reported in a US-based study published in 1993. This study, set in a primary care setting, reported prevalence estimates of 0.3%, 0.4% and 1.0% based on the CDC, British and Australian case definitions respectively [30].

Higher rates have been reported in community-based studies. In one study conducted in the US, using several approaches of case ascertainment including the CDC criteria, prevalence was estimated to range between 75–267/100,000 population [31]. A second, large community-based study using screening, followed by self-report questionnaires, psychiatric and medical examinations, reported a prevalence rate of 0.42% (95%CI 0.29–0.56) [32]. A third community-based study – which ascertained cases based on interviews on the duration and impact of fatigue, based on the Fukuda criteria – reported 0.2% of participants were classified as being CFS-like [33]. These higher rates are likely to be related to case ascertainment being conducted by researchers/medical practitioners who have expertise with CFS. In a systematic review in which meta-analyses were conducted, prevalence estimates based on diagnostic criteria were reported as follows: 0.89% (95%CI 0.60–1.33) using the CDC-1994 case definition criteria, 0.17% (95% CI: 0.06–0.49) using Holmes criteria, and 1.41% (95% CI: 0.68–2.93) using the Oxford criteria [11]. Variations in estimates from these previous studies further suggests the possibility of underestimation even when strict case definition criteria are applied.

In our study, we reported higher crude prevalence estimates for females compared to males, with rates between 130.0–141.4/100,000 for females and 50.9–57.5/100,000 for males. This difference was not driven by a substantially higher primary care attendance rate by females, as 54.7% of all MedicineInsight patients are female [34]. This is consistent with other research that has reported higher prevalence in females. For example, in a recent meta-analysis, the authors reported prevalence estimates of 1.36% for females and 0.86% for males [9]. Similarly, a US based study also reported a higher rate in females at a ratio of 11.2:1.0 [10].

We also estimated crude prevalence by age groups and found rates to be higher in age group 50–59 years followed by 40–49. A small number of studies have reported on age: the previous Australian study reported a higher rate for participants aged 40–49 years (110 per 100,000 population) [14]. A second study conducted in three regions of England reported a mean age of 49.3 years [7]; whilst in the systematic review by Lim et al., a mean age of 40.4 ± 7.7 years was reported [11]. The observed variations in the mean age reported in these studies may be a result of the different case definition criteria or diagnostic criteria: each diagnostic criterion has a limit to the scope it covers and the level of sensitivity it addresses. The choice of the diagnostic criterion determines who gets enrolled as part of the study population. Another very important factor is the size of the study population as the larger the sample size the closer the mean age will be to the true age of onset for ME/CFS in the general population.

In all years there were higher prevalence rates for inner regional areas than for people residing in major cities, remote or very remote regions. The ABS defines inner regional areas as “areas where geographic distance imposes some restriction upon accessibility to the widest range of goods, services and opportunities for social interaction” [19]. During discussions with the PAG, several members noted that they had moved from a major city to a smaller town due to lower living costs and improved lifestyle within the constraints of ME/CFS. Of note, qualitative interviews and focus groups with people from rural and regional areas discussed the lack of specialist primary healthcare services in their area, and even the lack of understanding regarding ME/CFS in the GP primary healthcare setting.

We did not observe any clear trends regarding IRSAD quintiles based on our quantitative analyses. This contrasts with a previously published study in the US found which reported higher rates amongst the least affluent groups [33]. This study was conducted in a community setting and involved telephone screening of a random sample of 8,004 households for ME/CFS-like symptoms. The authors reported that ME/CFS was more prevalent amongst households with a combined annual income of less than USD40,000. As our prevalence estimates are based on attendances in the primary care setting, individuals experiencing socio-economic disadvantage may be under-represented. Whilst Australia has universal health insurance, substantial resources such as time, health literacy and money are frequently required to seek a diagnosis of ME/CFS. This theme was identified in the focus groups and interviews: with substantial financial costs to patients and carers associated with the extended time taken for a diagnosis to be made, with visits to many types of health care providers and investigations. In turn, this may underestimate the true prevalence rate amongst individuals with fewer resources available to receive an ME/CFS diagnosis.

A key strength of this study was the size and national coverage of the national NPS MedicineWise MedicineInsight dataset, along with the robust approach to defining cases. There are also limitations of our study that require consideration. Our approach to case definition meant that when an ME/CFS patient attended an appointment for an ME/CFS symptom such as dizziness/orthostatic intolerance and the encounter reason was recorded as such, in the absence of another encounter with any of the pre-specified ME/CFS, the patient was not counted as a prevalent case for that year of interest. This will contribute to what we expect is an underestimate of the true prevalence of ME/CFS in primary care settings. In addition, considering the non-uniformity and lack of generalizability of diagnostic criteria for ME/CFS, some of the nomenclature used in communicating diagnosis may not necessarily connote the right intention of the GP. We were also unable to ascertain the diagnostic acumen of the GPs or the presence of biases which might lead GPs to be more or less likely to record a diagnosis of ME/CFS. Originally we had planned to conduct a clinical audit to address this, however due to COVID-19 and the resulting travel restrictions and primary care clinics declining research activities that were not core-business, this was not possible. The most commonly used case definition criteria in Australia are the Fukuda and the Canadian Consensus Criteria; therefore, based on insights from our qualitative analysis, we assume that most cases in our datasets are likely to conform to these criteria. It is important to note, however, that the representativeness of the participants in the qualitative aspects of this study is not known. Another key limitation of our study is the identification of cases from primary care data that largely precluded people with ME/CFS who are housebound or bed bound. In Australia, approximately 96% of consultations are conducted within the practice consulting rooms, rather than within the patient’s homes or at residential aged care facilities. To highlight this, we conducted the qualitative study which included bed-bound participants, to provide more in-depth evidence on the under-estimation of ME/CFS prevalence.

## Conclusion

This study provides up-to-date prevalence estimates for ME/CFS in the Australian primary care setting. It also presents qualitative evidence from patients and carers that highlights the limitations of our quantitative estimates. It is likely that at least 20,000 Australians were living with ME/CFS in 2019. To address the likely underestimate of prevalence, more accurate prevalence rates could be calculated in future prospective studies that use robust diagnostic criteria for case ascertainment in the community setting. In turn, quantification of the burden of disease associated with ME/CFS can be used to inform health policy and planning and prioritise research funding for this understudied condition.

## Supplementary Information

**Additional file1: Appendix 1.** Potential reasons for an underestimate of prevalence of ME/CFS in the primary care setting.

## Data Availability

The quantitative data that support the findings of this study are available from NPS MedicineWise but restrictions apply to the availability of these data, which were used under license for the current study, and so are not publicly available. The deidentified qualitative data are not publicly available due to confidentiality and the sensitive nature of these data, but are available from the corresponding author on reasonable request.
