# Supplementary material for: Prevalence of myalgic encephalomyelitis/chronic fatigue syndrome (ME/CFS) in Australian primary care patients: only part of the story?
Source: BMC Public Health. 2022 Aug 9;22:1516. doi: 10.1186/s12889-022-13929-9 (PMC9363145; doi:10.1186/s12889-022-13929-9)
Supplement: Supplementary file 1 — Additional file1: Appendix 1. Potential reasons for an underestimate of prevalence of ME/CFS in the primary care setting. [file 12889_2022_13929_MOESM1_ESM.pdf]

APPENDIX 1: Potential reasons for an underestimate of prevalence of ME/CFS in the primary care setting

A. The case definition criteria used for the MedicineInsight data analyses included the following terms: “myalgic encephalomyelitis”, “chronic fatigue syndrome”, “ME”, “CFS”, or “ME/CFS”. As a result, patient encounters with other terms that can be synonymous with ME/CFS would result in exclusion of the patient as a case. Examples of these terms were provided by the Patient Advisory Group:

- Systemic Exertion Intolerance Disease (SEID);
- Chronic Fatigue and Immune Dysfunction Syndrome (CFIDS);
- Post Viral Fatigue Syndrome (PVFS);
- Burn Out;
- Chronic Fatigue; and
- Chronic Lyme’s disease.

As noted in the qualitative evidence, a diagnosis of ME/CFS can take several years. As such, patients going through this process will not be counted as cases.

B. Some patients may have given up on seeking help from the medical healthcare system.

C. Some patients, the housebound and bedbound, are unable to present in the ambulatory care setting.

D. For patients who have ME/CFS, the encounter may be recorded for the presenting symptom, for example:

- Postural orthostatic tachycardia syndrome (POTS);
- Irritable bowel syndrome (IBS); and
- Sleep disorders such as narcolepsy or multiple chemical sensitivities (MCS).

Anecdotal reports suggest that patients may be given an alternate diagnosis to access pharmaceutical treatments at lower cost. For example, a diagnosis of narcolepsy will grant a patient subsidised access to armodafinil ($41.30); alternately off-label supply will be more costly ($256).

E. ME/CFS not diagnosed, and the patient has been given as incorrect diagnosis, for example, of:

- Depression;
- MS;
- Lupus; and
- Sjogren’s Syndrome.
